# What are the informational pathways that shape people’s use of cannabidiol for medical purposes?

**DOI:** 10.1186/s42238-021-00069-x

**Published:** 2021-05-06

**Authors:** Marco A. Zenone, Jeremy Snyder, Valorie A. Crooks

**Affiliations:** 1 Faculty of Health Sciences, Simon Fraser University, 8888 University Dr, Burnaby, British Columbia V5A 1S6 Canada; 2 Department of Geography, Simon Fraser University, 8888 University Dr, Burnaby, British Columbia V5A 1S6 Canada

**Keywords:** Cannabidiol, Crowdfunding, Cannabis, Decision-making, Medical treatment

## Abstract

**Background:**

Cannabidiol (CBD) is commonly used to manage symptoms in conditions and diseases for which there is limited clinical research for its application. How consumers arrive and decide to use CBD for medical treatment, despite lacking clinical evidence, is largely unknown. In this paper, we seek to identify the informational pathways through which consumers arrive at CBD for medical purposes.

**Methods:**

GoFundMe.com campaigns fundraising to purchase CBD between June 2017 and May 2019 were collected using the Crowdfunding for Health Research Portal (CHRP). Product descriptions were thematically analyzed to determine pathways leading to incorporation of CBD into medical treatment. Campaign characteristics such as fundraising ask, funding received, location, campaign title, description, Facebook shares, and number of donors were recorded. Specific medical uses of CBD proposed in campaigns were tabulated.

**Results:**

The study identified 164 crowdfunding campaigns primarily from the USA (*n*=159), with several from Canada (*n*=5). The campaigns requested $2,219,284.24 (median, $7000) and raised $610,612.87 (median, $1805) from 6825 donors (median, 26). Many campaigns asked for other treatments or illness-related costs not specific to CBD. The campaigns were shared 42,299 times on Facebook (median, 156 shares). Three informational pathways were identified leading to incorporation of CBD into medical treatment, which were self-directed research (*n*=149), recommendations from a trusted care provider (*n*=36), and/or experiential insights shared by someone associated with or influencing the crowdfunders personal network (*n*=30). The proposed uses of CBD were for cancer (*n*=96), seizure-inducing diseases/conditions (*n*=48), other/unspecified (*n*=6), joint/inflammatory diseases (*n*=6), mental health disorders (*n*=3), nervous system diseases (*n*=3), and autoimmune diseases (*n*=2).

**Conclusions:**

Our results suggest that consumers crowdfunding come to CBD through internally motivated reasons versus exposure to advertisements or other forms of marketing. Campaign beneficiaries generally had an unmet medical need that other forms of treatment were not satisfying. Then, through one or more of the informational pathways identified, CBD is considered a potential solution.

## Background

Cannabidiol (CBD) is a non-psychoactive cannabinoid found in the cannabis family of plants (Davis 2019). The use of CBD for medical purposes has increased dramatically over the past 5 years. In 2014, CBD sales in the USA were $108 million, increasing to $1.9 billion in 2020 (Mikulic 2019). Increases in CBD medical usage are in part due to the declassification of CBD products derived from hemp containing less 0.3% tetrahydrocannabinol (THC) as a controlled substance in the USA due to the signing of the 2018 Farm Bill and legalization of cannabis in Canada (Banda 2018; Government of Canada 2018). Limited scientific evidence for CBD exists for uses such as pain management, depression, cancer treatment, osteoarthritic disorders, and mental health conditions (MacKeen 2019; Rabin 2019; Lewis 2019; Khan et al. 2020; Batalla et al. 2019; Ghabrash et al. 2020; Broyd et al. 2016; Schubart et al. 2014; Blessing et al. 2015; Lee et al. 2017). There have been numerous studies with promising results for CBD usage for treatments such as epilepsy and related conditions (Lattanzi et al. 2018; Iseger and Bossong 2015; Osborne et al. 2017; Honarmand et al. 2019; Ramer et al. 2012; Hunter et al. 2018; Maayah et al. 2020; Junior et al. 2020; Elliott et al. 2020; Klotz et al. 2018). Recently, the US Food and Drug Administration (FDA) approved Epidiolex—a CBD oral solution for advanced epilepsy treatment (United States Food and Drug Administration 2018).

Despite advancing research, academics and news outlets have expressed concerns over hype—understood as extreme promotion of a medical treatment or service above what is justified by evidence of efficacy—surrounding CBD (Caulfield 2019; Friedman 2018; Petrow 2019; Eisenstein 2019; Schraer 2019; Adams 2019). Represented often as a cure-all and marketed with non-evidence-based or exaggerated efficacy claims, current uses of CBD do not align with extant evidence of safety and efficacy (Caulfield 2019). While regarded as a relatively harmless substance, the hype surrounding CBD is concerning because persons may spend substantial amounts of money and choose to forego effective treatment for unproven CBD treatments, relying on unproven and potentially inaccurate claims (MacKeen 2019; Burns 2018).

Limited literature exists exploring why people are increasingly trying CBD for a range of ailments. Media discourse suggests several possible reasons. First, anecdotal testimonials, often from celebrities or other persons of influence, are highly visible and frequently come from what are perceived to be trustworthy sources. These testimonials document the efficacy of CBD for numerous health purposes. Claims range from pain relief to chronic disease treatment such as cancer (25 Famous Celebrities and Athletes Who Use or Endorse CBD, 2019). For example, the National Football League (NFL) player Rob Gronkowski has advocated that CBD is the most effective treatment for his chronic pain resulting from his football career (Chidley-Hill 2019). Other testimonials include celebrities such as Kim Kardashian using CBD for anxiety relief and Duchess Camilla of the UK stating it is ‘fantastic’ when visiting a London farmers market (Parker 2019; Turril 2019).

Second, CBD is generally safe and not psychoactive; therefore, the potential negative consequences of trying CBD are perceived to be slight and its use is seen to be socially acceptable (MacKeen 2019). The non-psychoactive properties of CBD allow for justification from a wide range of users who might not openly use products containing THC or other psychoactive molecules. However, the FDA warns CBD may cause liver injury, impact fertility in males, or interact with other drugs, and that the composition, quality, and safety of CBD products vary by manufacturer (United States Food and Drug Administration 2020).

Third, CBD is available in formats friendly to consumers and thus attractive and for experimentation. For example, CBD is available in popular foods (gummies, cheeseburgers), hygienic products (toothpicks, shampoos), and a wide variety of other products (MacKeen 2019; Petrow 2019; Williams 2018; Gavura 2019). While these routes to trying CBD may partially explain experimentation, the underlying personal reasons people are trying CBD are likely more complicated and contextualized. Thus, to inform public policy and understand the hype surrounding CBD for medical purposes, additional research is needed that considers the personal experiences and narratives of individuals using or considering trying CBD.

An effective strategy to better understand the informational pathways that shape decisions regarding CBD usage is to examine medical crowdfunding campaigns. Crowdfunding platforms allow users to host fundraising campaigns for medical purposes and share these campaigns on social media. These campaigns often describe the personal and medical context of the fund recipient and offer explanations for their proposed use of funds. The content is unsolicited, therefore allowing for unprompted narratives detailing the point of view of the fundraiser. Researchers have used crowdfunding data to explore fundraising needs and motivations for other groups such as transgender communities, people with cancer accessing complementary and alternative treatments including cannabinoids, and those seeking stem cell treatment for autism (Barcelos and Budge 2019; Zenone et al. 2020; Snyder et al. 2020; Snyder and Turner 2020). CBD is costly, typically not covered by public or private insurance, and unaffordable to many, therefore providing conditions for online fundraising for CBD using crowdfunding platforms . Thus by using crowdfunding campaigner narratives, this analysis seeks to augment existing research on factors promoting the use of CBD and identify specific informational pathways these individuals go through prior to arriving at a decision to utilize CBD for medical purposes.

## Methods

Crowdfunding campaigns on GoFundMe.com containing the terms ‘cannabidiol’ or ‘CBD’ were retrieved using an automated web scraper tool. The tool, the Crowdfunding for Health Research Portal (CHRP), is a database of GoFundMe campaigns that includes campaign characteristics such as title, category, location, funding pledged, funded received, Facebook shares, number of donors, as well as the description and updates. CHRP has collected campaigns posted to the GoFundMe online sitemap from April 2019. Our search retrieved 1547 GoFundMe campaigns referencing cannabidiol or CBD. GoFundMe categories not related to medical uses were removed from these results (*n*=1101 remaining) and a two-year cut off (June 2017 to May 2019) implemented (*n*=727 campaigns remaining). We chose these date parameters to coincide with approximately one year before and after the initial cannabis legalization date of July 1st, 2018, in Canada and ongoing cannabis legalization discourse in the USA (Scotti 2018; Cobb 2019). Geographic parameters limited campaigns to only Canada and the USA for these reasons, leaving 596 campaigns. The first author then reviewed each campaign to determine inclusion. Campaigns were included if the campaign organizer was directly crowdfunding for CBD for a medical purpose in humans. The second author decided inclusion for campaigns flagged by the first author. After reviewing each campaign, 164 remained. Campaigns were excluded for not crowdfunding for CBD (*n*=372), using CBD for an animal (*n*=37), legal issues related to CBD (*n*=10), campaign text not available in English (*n*=6), or outside of the geographic inclusion area (*n*=7).

Each author independently reviewed 30 campaign descriptions and met to discuss how the campaigners decided to utilize CBD as a potential treatment option. It was through this process that a focus on informational pathways emerged. Three dominant pathways were identified that led campaigners to try CBD: (1) self-directed research, (2) recommendation by a trusted care provider, and/or (3) experiential insights offered from someone associated with or influencing the personal network. The first author independently assigned each campaign to a dominant, and in some cases also secondary, pathway in a spreadsheet and recorded the medical condition inspiring crowdfunding for CBD. The second and third authors each audited 25% of campaigns to ensure consistency and confirm the interpretation of the informational pathways. Any disagreements identified by the second and third authors were resolved through discussion. Although campaigns were posted without an expectation of privacy, we have protected the details of campaigners as much as possible by avoiding any identifying descriptors.

## Results

The 164 included campaigns requested $2,219,284.24 (median, $7000) and raised $610,613.87 (median, $1805) from 6825 donors (median, 26). Many campaigns asked for other treatments or illness-related costs not specific to CBD. The campaigns were shared 42,299 times on Facebook (median, 156 shares). Most campaigns originated in the USA (*n*=155), with few from Canada (*n*=9). The proposed uses of CBD were for managing the symptoms of, or seeking a cure for, cancers (*n*=96), seizure-inducing diseases/conditions (*n*=48), other/unspecified conditions (*n*=6), joint/inflammatory diseases (*n*=6), mental health disorders (*n*=3), nervous system diseases (*n*=3), and autoimmune diseases (*n*=2) (see Table 1). In the most prevalent proposed use, cancer, CBD was proposed for curative or primary treatment (*n*=57), pain/symptom relief from cancer and cancer treatment (*n*=24), enhancing conventional treatment (*n*=11), and unspecified uses in 4 campaigns. For the second most prevalent use, seizure-inducing diseases/conditions, all campaigns (*n*=48) were to prevent seizures. Table 1 provides the intent of using CBD for the other diseases and conditions.

**Table 1:** Disease or condition proposed for CBD usage on GoFundMe.com by intended outcome, Facebook shares, donors, total requested, and total received

Disease or condition	Intended outcome	Facebook shares total	Number of donors total	Total requested in $US	Total received in $US	Total campaigns
Cancer	Enhance conventional treatment	5146	888	236,237.7	95,862.21	11
	Pain/symptom relief	4049	843	233,476.22	74,227.7	24
	Treatment/cure	18,023	3065	1,018,765.54	281,692.77	57
	Unspecified	514	76	45,000	12,750	4
	**Total**	**27,732**	**4872**	**1,533,479.46**	**464,532.68**	**96**
Seizure inducing diseases/conditions	Seizure relief	10,937	1306	466,397.35	96,814.44	48
	**Total**	**10,937**	**1306**	**466,397.35**	**96,814.44**	**48**
Other/unspecified conditions	Pain/symptom relief	587	109	47,555	6753	4
	Symptom/pain relief and seizure relief	101	13	1500	950	1
	Unspecified	21	9	50,000	570	1
	**Total**	**709**	**131**	**99,055**	**8273**	**6**
Joint/inflammatory diseases/symptoms	Pain/symptom relief	638	208	52,401.62	12,591.18	4
	Treatment/cure	772	72	12,000	7547	2
	**Total**	**1410**	**280**	**64,401.62**	**20,138.18**	**6**
Nervous system diseases	Pain/symptom relief	69	21	4000	1665	1
	Treatment/cure	419	50	19,450.81	4884.57	2
	**Total**	**488**	**71**	**23,450.81**	**6549.57**	**3**
Mental health conditions and disorders	Pain/symptom relief	159	15	11,500	1760	2
	Treatment/cure	430	82	16,000	9365	1
	**Total**	**589**	**97**	**27,500**	**11,125**	**3**
Autoimmune disorders	Treatment/cure	434	68	5000	3180	2
	**Total**	**434**	**68**	**5000**	**3180**	**2**
**Grand total**		**42,299**	**6825**	**2,219,284.24**	**610,612.87**	**164**

The table is a summary of crowdfunding campaigns on GoFundMe.com fundraising cannabidiol for medical usage

Self-directed research (*n*=149) was the most commonly observed pathway to trying CBD. This informational pathway is characterized by the campaigner or a close loved one undertaking significant self-directed research to identify symptom management strategies or cures in the context of having limited, ineffective, or no other options to support their disease or condition. This self-directed process of reviewing websites and other informational sources then led individuals to pursue the usage of CBD. This pathway is seen predominantly in cancer and seizure-inducing diseases and conditions where no cure exists or symptoms were debilitating. For example, the family of a young child with frequent seizures found CBD through self-research after repeated unsuccessful management attempts: ‘We have done research on CBD oil and it is something we are interested in and believe will help stop the seizures’. A second campaign describes a husband completing significant research on alternative treatments after being told his wife’s cancer is incurable and thus arriving at CBD: ‘My wife has small cell cancer of the pancreas. We were told by doctors that there is nothing more they can do. I have been investigating alternative therapies and they look very promising’. Campaigns describing this informational pathway were additionally characterized by those who came to CBD from a hope or desire to treat their disease or condition using natural or alternative options. Often, citing the perceived harms of Western medicine or holding beliefs that the body is adequately prepared to heal itself. For example, a person with non-small neuroendocrine cancer rejected chemotherapy, radiation, and surgery in favour of a self-researched protocol incorporating CBD: ‘I decided to follow a natural treatment protocol which included raw juicing, buying an oxygen system, daily supplements, CBD oil, lots of prayer, and travelled near and far seeking natural treatments’.

Some campaigners tried CBD as a result of a recommendation from a trusted care provider as a key information source (*n*=36). Initial recommendations for CBD came from both medical practitioners such as physicians, as well as alternative practitioners such as naturopaths. Such individuals formed the basis of this informational pathway. Physicians often recommended CBD to help control seizures. For example: ‘A new neurologist along with another one of her doctors has recommended that we start [recipient] on CBD oil. The CBD oil has the potential to be life-changing for [recipient]. We are hopeful that it could help with her seizures’. Alternative practitioners were observed in some cases advising patients for natural cancer regimens, usually in the context of having no other options. For example, a person with stage 4 breast cancer was crowdfunding treatment prescribed by a naturopathic oncologist, which included ‘high doses of RSO, CBD, Vitamin IV therapy’. There were numerous instances of a trusted care provider being approached by a person or their caregiver seeking to try CBD oil but wanting to first ensure its safety or efficacy, therefore acting as gatekeepers. For example: ‘[Rachel]’s family research alternative methods to try to control her seizures and asked the doctor about CBD. He was immediately on board with the ideas and gave her a recommendation for it’.

Experiential insights from someone associated with or influencing the crowdfunders personal network drew some people to CBD (*n*=30), and this forms the basis of the third informational pathway identified. CBD recommendations came from a person in the immediate social network of the person or their caregiver, including family, friends, colleagues, and acquaintances. Other recommendations came from someone associated with but not in the personal network such as strangers (online or in-person), via social media messages, or from shared online testimonials. An example of someone within the social network is a daughter who did online research and recommended to her mom to try CBD: ‘After doing much research since the beginning of our Mother’s diagnosis she [daughter] read on about Cannabis oil and Rick Simpson’s oil and about people healing themselves of tumours and cancer by ingesting high levels of THC and CBD’. Recommendations from outside the personal network, such as strangers, often reached came through social media or traditional media such as viewing testimonials on television programs. For example, a family struggling to control the seizures of their youngest daughter saw an interview on CNN (an American news network) about a girl who was experiencing over 300 grand mal seizures per week and who found relief from CBD. The parent responded by making it a mission to try CBD. In a second case, a family is reached out to on Facebook with information that CBD is a viable option for cancer treatment: ‘I had some wonderful ladies private message me that are going through the same thing, have started CBD and some are in remission. There is hope!’. The exchange led to the family trying and prioritizing CBD treatment.

## Discussion

Our findings suggest that crowdfunders are basing their decisions on CBD use primarily from information they have compiled from their own research to address a serious health issue, usually not accompanied by medical advice. That the most commonly observed informational pathway to CBD use is self-directed research suggests that most campaigners were satisfied in-part by the evidence or testimonials they find from their online search strategies. Similarly, those referencing an experiential insight from another person who has had success with CBD found the testimonials compelling and were at least in-part satisfied with the anecdotal information provided. Only a small number of crowdfunders in our sample detailed having consulted a qualified medical provider in their decision.

Campaigners often had few to no perceived effective options for managing their health, often describing terminal diagnoses, significant pain, uncontrolled symptoms from numerous diseases, and/or other issues requiring intervention. Some crowdfunders mentioned emerging research that CBD may support their condition, as well as many others. For some of the conditions listed, such as cancer, there is early, premature research for its possible treatment and therapeutic use (Honarmand et al. 2019; Zhang et al. 2019; Dariš et al. 2019). The perception of untapped medical potential made CBD an attractive option as there was little to nothing for campaigners to lose from experimentation. Other studies have found that many people believe there is not yet discovered medical potential (Corroon and Phillips 2018; Tran and Kavuluru 2020). Amplifying its attractiveness to crowdfunders, many viewed CBD as a safe, natural substance, absent of psychoactive properties. News reporting commonly comments on such perceptions (Rabin 2019; Friedman 2018; Halperin 2018; Ramanathan 2018). Thus, to crowdfunders, CBD appears to offer a harmless chance of treatment or symptom relief in the absence of other options.

A significant finding from our study is that those fundraising for CBD on GoFundMe.com usually were motivated and pursue CBD through their own initiative versus exposure to advertisements, the advice of qualified medical professionals, or the opinion of alternative medical practitioners. Policy responses to regulate or ensure the appropriate usage of CBD need to consider that those using CBD for medical purposes—whether for pain relief or for treatment of serious illnesses—were usually people seeking information and arriving at informational pathways from outside of the medical hierarchy. To promote retrieval of information from qualified sources, several evidence-based strategies are available. These include health literacy interventions, incorporation of patient perspectives into treatment decisions, careful media dissemination of new study findings to avoid sensational reporting, online fact-checking, and social media campaigns (Trethewey 2020; Hawke et al. 2019; Siminoff 2013; Chou W-YS and Klein 2018).

There were important differences between the informational pathways of those trying CBD for epilepsy versus other health conditions referenced in the reviewed crowdfunding campaigns. There is clinical acceptance for the use of CBD for epilepsy, primarily for the management of seizures (United States Food and Drug Administration 2018)^.^ Those trying CBD for this reason often incorporated the advice of a medical practitioner into their treatment decision, as opposed to other uses. Those utilizing CBD for other symptoms and diagnoses seemingly made the decision with limited or no clinical acceptance. For example, those incorporating CBD for cancer into their treatments commonly presented narratives where their healthcare system did not have any curative options left for them or the prescribed treatment was ineffective. The usage of CBD was seen as an alternative option to those offered by a medical system that had abandoned them. In the most extreme cases, foregoing accepted medical treatments in favour of CBD or relying on CBD for consequential diseases such as cancer. This finding underscores the importance of policymakers and medical bodies to provide and disseminate health literacy materials for the scientifically-supported uses of CBD.

Several research areas were identified that need further exploration. First, the information sources that campaigners in our study referenced—blogs, product descriptions, and information websites—need further study so we can understand their content. This can inform what messages are being transmitted that lead to CBD experimentation. Second, our study could not capture the interactions between campaigners and the in-person conversations they had with those who work at cannabis dispensaries (both legal and non-legal) or other providers. Numerous campaigners in our study reference that they were guided from knowledgeable persons such as cannabis retail workers in decision-making around CBD, including dosing, product selection, and method of administration. The lack of information available around such interactions and subsequent product selection, including CBD product form, require further investigation. Future research should incorporate qualitative interviews into those who help prospective CBD consumers initiate and guide treatment.

Our study has several limitations. The data collected is self-reported and the accuracy of campaigns was subject to the truthfulness of campaigners. There may be information selectively omitted to make a compelling case for donations. Second, our study does not capture all fundraising of CBD products for medical purposes. Our search strategy, while robust, does not capture CBD products which might go by a different name.

## Conclusion

This study explored the informational pathways used by those fundraising for CBD on GoFundMe with the intention of incorporating CBD into their medical treatment. After identifying 164 campaigns using CBD for medical purposes on GoFundMe.com, we discerned that most potential or current CBD users arrived at the decision the result of three pathways: self-directed research, a recommendation from a trusted care provider, or from the anecdotal experience of another. The medical uses of CBD were for a variety of purposes, with most being for cancer or epilepsy. CBD filled an urgent void or need for most campaigners—in that they had ineffective or no options, did not trust the other treatment options available, or wanted a natural treatment option. This information should help policy makers and patient advocates to craft targeted interventions for users of CBD, including combatting specific sources and types of misinformation. Future research is needed to understand which self-directed sources of information potential CBD users find and use to inform their treatment decisions.

## Data Availability

The datasets used and/or analysed during the current study are available from the corresponding author on reasonable request.

## Abbreviations

CBD: Cannabidiol
THC: Tetrahydrocannabinol
FDA: Food and Drug Administration
NFL: National Football League
CHRP: Crowdfunding for Health Research Portal
CNN: Cable News Network
